# Elucidating the emotional persona in the Romanian university students’ academic discourse: a corpus-based exploration

**DOI:** 10.3389/fpsyg.2024.1514795

**Published:** 2025-01-20

**Authors:** Diana Paula Dudău, Madalina Chitez, Florin Alin Sava

**Affiliations:** ^1^Department of Psychology and Psychotherapy, Faculty of Psychology, Titu Maiorescu University, Bucharest, Romania; ^2^Education Research Unit, National Center for Policy and Evaluation in Education, Bucharest, Romania; ^3^Department of Modern Languages and Literatures, Faculty of Letters, West University of Timișoara, Timișoara, Romania; ^4^Department of Psychology, Faculty of Sociology and Psychology, West University of Timișoara, Timișoara, Romania

**Keywords:** academic writing, emotions, automatic language analysis, ROGER corpus, LIWC, cultural influences, multilingual higher education, cross-linguistic differences

## Abstract

**Introduction:**

Despite growing global interest in the emotional dimensions of academic writing, Romanian academic discourse remains underexplored, particularly in multilingual contexts. This study addresses this gap by analyzing a bilingual corpus of texts written in Romanian (L1) and English (L2) across various disciplines and genres. It aims to uncover emotional dimensions conveyed through linguistic markers, exploring how language, culture, and academic context shape students’ writing styles. Romania’s historical and social emphasis on formality, hierarchy, and indirectness in communication serves as a backdrop for examining these dynamics.

**Method:**

A corpus-based approach was adopted, utilizing the Linguistic Inquiry and Word Count 2015 (LIWC2015) tool to analyze linguistic and emotional markers. The bilingual ROGER corpus, containing texts from nine Romanian universities spanning multiple disciplines and genres, served as the dataset. Advanced data analysis techniques included supervised machine learning for language classification, network analysis to explore interactions among linguistic features, and cluster analysis to detect discipline- and genre-specific linguistic patterns.

**Results:**

The findings reveal distinct emotional patterns between Romanian and English academic writing. Romanian texts exhibit a higher degree of formality and indirectness, while English texts reflect greater assertiveness and personal engagement. Additionally, the Romanian corpus demonstrates less linguistic cohesion and a broader range of writing styles. Genre- and discipline-specific trends also emerge, with English coursework and analytical writing, predominantly from social sciences, displaying more personal and emotional expression than research-focused texts. In contrast, the Romanian corpus, characterized by a third cluster, presents less clear-cut patterns: humanities texts span both emotionally expressive and neutral tones, while research and academic papers frequently exhibit an achievement-oriented or entrepreneurial style, though a significant subset also reflects a highly disengaged profile.

**Discussion:**

By integrating machine learning, network analysis, and automatic language analysis, this study offers a novel perspective on how language, genre, and discipline-specific conventions shape emotional expression in academic writing. The results suggest that the Romanian students’ emotional personas in academic writing are influenced by all these factors, potentially shaped by the cultural norms of the second language, providing insights for teaching academic writing in multilingual settings.

## 1 Introduction

Academic writing is not only an educational skill that demonstrates the students’ abilities to present, analyze, and communicate disciplinary content, but it also offers a window into their emotional and psychological states. This is particularly relevant for exploring whether features of academic discourse within a specific group reflect the emotional persona of that group. Pennebaker et al. (2014) demonstrated that subtle linguistic choices, such as function words in college essays, can reveal underlying cognitive and emotional processes, offering valuable insights into students’ emotional engagement and academic success. In the case of Romanian university students, no prior research has been conducted to systematically identify the emotional prompts embedded in their academic discourse.

Investigating how Romanian students’ writing reflects their emotions and attitudes is especially relevant, as it mirrors the societal shift from the communist era, where there was a tendency to conceal and repress thoughts, to the democratic period, where expressing opinions is both allowed and valued (Doroholschi et al., 2018). In addition, the introduction of additional writing cultures (Chitez and Kruse, 2012) into education, such as English-language norms, can contribute to significant changes in how students construct and express their identities. Exposure to different linguistic and rhetorical standards, particularly those that prioritize critical thinking and open discourse, encourages students to adopt more expressive and analytical approaches to academic writing. This cultural and linguistic shift not only broadens the students’ communicative skills but also requires them to address the intricacies of expressing personal and emotional nuances within academic frameworks.

Despite the growing global interest in the emotional dimensions of academic writing, Romanian academic discourse remains underexplored. The legacy of collectivist educational practices from the communist era, which often emphasized conformity and formality, may have inhibited emotional self-expression. Moreover, adapting to international writing norms potentially without adequate pedagogical support might pose additional challenges for Romanian students, especially because all Romanian teachers and professors in activity since the fall of the communist regimen to present were educated in those times or were born right after the 1989 revolution. Thus, this dual tension – between preserving cultural identity and adopting global standards – adds complexity to elucidating emotional personas in students’ writing and brings forward interesting questions.

Existing literature on the linguistic features of Romanian academic writing has primarily focused on phraseology (Chitez et al., 2021; Dincă et al., 2024; Muresan et al., 2022), argumentation (Tucan et al., 2020), and the development of computational resources such as the Romanian Academic Word List (Ro-AWL) (Bucur et al., 2022) and the Romanian Phrasal Academic Lexicon (ROPAL) (Chitez et al., 2021). These studies have offered valuable perspectives on both novice and expert academic writing, identifying key linguistic features that shape Romanian academic discourse. Furthermore, contrastive analyses between Romanian and English academic writing datasets have revealed distinctive characteristics of the Romanian writing style, particularly in how argumentation is structured and phraseological units are employed (Manda and Chitez, 2022; Bercuci and Chitez, 2023). However, in previous Romania-specific studies, emotional and psychological elements are often overlooked despite their relevance to both academic performance and the understanding of larger societal values (Williams, 2017). This leaves a significant gap in understanding how Romanian students’ linguistic choices reflect their emotional personas, and addressing this niche is crucial for developing targeted educational interventions in today’s world shaped by globalization.

The relevance of the linguistics-driven psychological approach to academic writing is manifold. For instance, the scrutiny of the students’ academic writing features, both linguistic and meta-linguistic, can reveal how they handle a disciplinary topic in terms of attitude: positively or negatively, assertively or hesitantly, confidently or with uncertainty (Hyland, 2005). The choice of words, sentence structure, and rhetorical strategies can indicate not only the level of subject mastery but also the emotional and psychological engagement of the writer (Hyland and Tse, 2007). For example, the use of modal verbs such as “might” or “could” may reflect hesitancy or a lack of certainty, while definitive language like “must” or “will” suggests assertiveness and confidence (Hyland, 2002). Additionally, variations in tone, whether formal, informal, or neutral, provide further clues to how students position themselves relative to the content, their audience, and the academic discourse community (Ivanič, 1998). These linguistic choices are often subconscious and can be influenced by a range of factors, including cultural norms, the perceived difficulty of the disciplinary field, and the expectations of the academic environment (Hinkel, 2001). Meta-linguistic features, such as hedging (e.g., “might,” “perhaps”), emphasis (e.g., “it is important to note”), boosters (e.g., “clearly,” “undoubtedly”), transition markers (e.g., “however,” “in addition”), frame markers (e.g., “first,” “finally”), and attitude markers (e.g., “unfortunately,” “interestingly”) play a crucial role in shaping academic writing by guiding the reader through the argument and indicating the writer’s stance (Hyland, 1998). These elements help students navigate complex arguments, signal their engagement with the topic, and manage the relationship with their readers (Morita, 2004). Through careful analysis of these features, educators can not only correlate linguistic usage with the students’ academic performance but also gain insights into their emotional and cognitive states, which are often interconnected with their writing decisions (Swales, 1990).

Adding to these complex aspects is the lack of validated tools for analyzing emotional and cognitive dimensions in multilingual settings that include Romanian academic writing. While there are many automatic language analysis tools capable of extracting emotional valence or contents from any text – for a review, see Eichstaedt et al. (2021) and Neuendorf (2017)—their application in the Romanian language remains problematic because most of them were built for English and the translation and validation process of such instruments is not straightforward. Therefore, examining psychological markers of Romanian academic writing is in its infancy, which shows a pressing need for studies that bridge this research gap, especially concerning the emotional personas of Romanian students.

Building on this significant niche, in our study, we aim to address precisely this unexplored topic of elucidating the emotional persona in the Romanian university student’s academic discourse. Using the Linguistic Inquiry and Word Count (LIWC) tool—the 2015 version developed by Pennebaker et al. (2015)—this research analyzes how Romanian students’ writing in their native language (L1) and English (L2) reflects emotional and cognitive dimensions across various disciplines. This comparison between L1 and L2 texts enables us to investigate how language influences emotional and psychological expression in academic discourse, exploring whether students demonstrate different emotional personas when writing in their native language versus a second language. Furthermore, we assess how discipline-specific writing conventions influence the use of emotional and cognitive language, highlighting how academic fields shape students’ linguistic and psychological expression. Through this, we aim to uncover both linguistic patterns and emotional markers that reflect the students’ academic and personal identities.

Thus, the novelty of this research lies in its dual focus on emotional personas and multilingual academic writing. By applying LIWC2015, a validated tool for linguistic and psychological analysis, to a corpus of Romanian-English student texts, this study pioneers a rigorous approach to examining psychological markers in Romanian academic discourse. LIWC2015 was selected for its ability to extract a variety of psychological contents, including emotional, cognitive, and motivational dimensions, which could offer a comprehensive view of the emotional persona reflected in the Romanian student group’s writing. Recently tested and validated for the Romanian language (Dudău and Sava, 2022), LIWC has proven its versatility in conducting detailed analyses of specific word categories (Kahn et al., 2007; Pennebaker et al., 2015). In addition, it features functionalities such as Keywords in Context (KWIC), which capture the nuanced aspects of academic writing (Tausczik and Pennebaker, 2010). By contextualizing specific words, LIWC captures the students’ strategies for expressing analytical thinking, confidence, and emotional tone in their academic work. At the same time, LIWC’s closed-vocabulary approach has shown immense research potential in comparative research across languages and disciplines (Kučera and Mehl, 2022). The Romanian LIWC also proved equivalence with other language versions, not only with the original English one (Dudău and Sava, 2021), allowing a future valid extension of our study to more intercultural comparisons of emotional personas emerging from language. Thus, in academic writing, where culture- and language-specific rhetorical choices are evident (Hinkel, 2002), LIWC provides powerful, user-friendly automated tools—a validated, multilingual dictionary and accompanying software—for systematically analyzing these differences. Its use in this study establishes a reliable foundation for investigating cross-linguistic and cultural variations in academic discourse, extending beyond Romanian and English, and further enhancing the rigor and relevance of our research.

In line with our objective to analyze the emotional persona in Romanian university students’ academic discourse, this study aims to address the following key questions:

1. What are the key features of Romanian students’ emotional personas as reflected in their academic discourse, particularly in terms of emotional expression (e.g., positive and negative emotions)?
2. How do these emotional personas differ between Romanian and English academic texts, and in what ways do these differences influence students’ writing in each language?
3. What discipline-specific and genre-specific emotional identity traits can be identified in Romanian university students’ academic writing?

## 2 Literature review

### 2.1 Emotional persona and academic writing: the path towards a socio-cognitive perspective

To reach an understanding of the complex dynamics between the writing process and the psychological factors correlated with it, research has undergone a dual model approach to writing: writing as a product (the final text) and writing as a process (the cognitive steps involved in creating that text). The beginnings of writing research focused on the perception of writing as a static outcome of the human mind. Early composition studies looked at text in terms of grammar, sentence accuracy, and formal structure without considering the cognitive processes associated with it. Writing was viewed in its developmental perspective as a progressive mastery of discourse types (Moffett, 1968) or as a craft that can be learned through applying linguistic rules and conventions (Murray, 1968). A writing research paradigm shift was signaled by Hairston (1982), who highlighted the need to view writing as a cognitive process since writing encompasses more than the mere application of linguistic rules. It involves complex mental stages such as idea generation, planning, drafting, and revising. Hairston (ibid.) argued that focusing solely on the final product neglects the recursive and dynamic nature of writing, where cognitive tasks interact with the writer’s evolving text. This perspective paved the way for process-oriented approaches in writing pedagogy. Since the 1980s, this approach, as pioneered by Hayes and Flower (1980) and further developed by Bereiter and Scardamalia (2013), has conceptualized writing as a multifaceted interaction between cognitive functions, like working memory and executive control, and linguistic skills. As a result, the process approach has become the most comprehensive framework for understanding the complexity of writing (Alves and Haas, 2012), emphasizing how writers engage with their ideas and text throughout the writing process. Such view has greatly influenced writing pedagogy, advocating strategies that promote critical thinking and problem-solving skills in all writing activities, including academic writing.

Building on cognitive sciences, research has increasingly recognized that the analysis of emotional personas in academic discourse is deeply rooted in socio-cognitive perspectives on language and identity formation. These perspectives emphasize that writing is not only a reflection of individual cognitive processes but also a product of the social contexts in which it is produced. This aligns with Bereiter’s (1980) view that the development of academic writing is closely linked to broader cognitive and developmental processes, such as social cognition and reflective thinking. Pohl (2007) further highlights that key factors such as enculturation into academic norms, acquisition of disciplinary knowledge, and mastery of writing skills are shaped by both cognitive and social influences. In this view, writing is not merely a cognitive process but also a form of participatory sense-making that emerges through interaction with the social environment and artifacts (Vygotsky, 1978; Rogoff, 2003). The socio-cognitive perspective underscores that writing development is intertwined with socialization into academic norms and identity formation, thereby reflecting both cognitive functions and the socio-cultural contexts that shape academic discourse (Bereiter and Scardamalia, 2013; Pohl, 2007).

Such insights highlight that academic writing goes beyond technical skills, being a reflection of the writer’s engagement with their social and intellectual environment. This perspective has expanded to include emotional personas as essential components of academic writing. Emotional personas are expressed through the writer’s tone, style, and rhetorical choices, revealing their interaction with both the subject matter and the audience. Several studies have demonstrated the importance of examining both linguistic style and emotional expression to gain insights into the students’ attitudes, confidence, and engagement. In a study on film reviews, Argaman (2010) demonstrated that emotions such as happiness or sadness are conveyed through linguistic choices, i.e., intensifiers, metaphors, and first-person pronouns, illustrating how these elements reflect the writer’s emotional engagement with the content. In the case of academic writing, the emotional burden is heightened by the demands of the academic environment. In studies such as Negri et al. (2020), researchers have identified linguistic markers that carry a high emotional charge. Words such as “fear,” “pain,” and “despair” are indicative of heightened emotional arousal, signaling deep emotional responses to a topic. Cameron et al. (2009) examine how emotions such as self-doubt, anxiety, and fear are intricately connected to the challenges inherent in the research process. These emotions are further intensified by the critical nature of academia, as students “felt emotions like self-doubt, anxiety, and fear as shaped by the practices of critique” (Cameron et al., 2009, p. 274). This underscores how the critical framework of academia amplifies the emotional struggles faced by novice writers (ibid.).

In addition to the emotional labor involved in receiving and responding to feedback (Carless and Boud, 2018), academic writing involves several key emotional dimensions. Writers must balance asserting authority and expressing humility through linguistic strategies such as hedging (Hyland, 1996), the use of personal pronouns (I/we) (Hyland, 2002), and tone (Ivanič, 1998). Building on this, Liu (2013) study on the use of *Appraisal* resources in academic writing reveals that emotions such as satisfaction and personal engagement are expressed through authorial *Affect* values, which serve to project a strong personal voice in argumentative writing. These emotional cues, while subtle, play a crucial role in shaping the academic writer’s identity and stance.

While international studies provide valuable insights, research on the socio-cognitive and emotional dimensions of academic writing in the Romanian context remains limited. As noted in the Introduction section, recent developments, such as the creation of resources like the Romanian Academic Word List (Ro-AWL) (Bucur et al., 2022) and the Romanian Phrasal Academic Lexicon (ROPAL) (Chitez et al., 2021), have advanced our understanding of linguistic features like phraseology and argumentation. However, the emotional aspects of academic writing have been insufficiently explored, leaving a significant gap in understanding how Romanian students express emotions and attitudes through language, particularly when transitioning from their native language to English or another foreign language.

In December 2024, we conducted a search on the Web of Science Core Collection using keywords in the title that signaled language use (e.g., “language,” “linguistic,” “discourse,” “writing,” “text,” “corpus,” “phraseology”) and keywords in the topic sections that reflected psychosocial variables (e.g., “persona,” “emotion*,” “cognit*,” “attitudes,” “motivation,” “values”). The search was filtered to include studies analyzing both Romanian and English languages and focused on recent publications (i.e., the last 10 years). This yielded 42 documents, many irrelevant to our research focus, with only 19 addressing students. Among these, only three papers were tangentially (not directly) relevant to our study. For instance, one paper presented two corpora of business expressions in English and Romanian containing annotated metaphors suitable for cross-linguistic comparisons (Ferrari and Boca, 2017). In another study, Cojocaru (2021) analyzed 50 classroom compositions, revealing that several discourse markers (mainly textual connectors) differ between native Romanian speakers and students learning Romanian as a foreign language. Additionally, Senar et al. (2024) explored how the fluid intelligence of Romanian immigrant students shapes the relationship between L1 knowledge and L2 performance in Spanish and Catalan, showing some lexical, morphosyntactic, and orthographic particularities when speaking in Catalan versus Spanish.

The literature search also identified a few papers more linked to our research, even though they did not contain the word “students” or were not focused on academic writing. In line with our intention to capture changes in emotional expression between languages, Bromberek-Dyzman et al. (2021), testing two groups of bilinguals (Polish-English and Romanian-English), revealed cross-linguistic effects on emotional word recognition. On a different note, Popescu (2017) analyzed the metaphorical language in Romanian and British business press, detecting some notable differences, especially in attitudes towards work, whereas Ghivirigă and Baciu (2015) showed that Romanian scientific texts demonstrate a preference for epistemic expressions through modal verbs, similarly to what previous literature on the English language indicated. Additionally, a few studies analyzed the discourse markers in Romanian and other languages to build a multilingual corpus (e.g., Silvano et al., 2022) or to investigate the linguistic borrowings in Romanian (e.g., Cojocaru, 2020), while others demonstrated efficient methods to establish a correspondence between English and Romanian metaphors or idioms despite socio-cultural footprints (Gogâță, 2023; Trantescu and Reiss, 2022). Finally, Boc (2020), in a theoretical paper, argued that language serves not only as a medium of communication but also as a determinant of national identity.

Despite these contributions, the lack of targeted research on how Romanian students’ emotional personas adapt across languages remains evident. Understanding these adaptations requires a deeper investigation into the interconnections between cognition, language, emotions, and socio-cultural factors, underscoring the need for studies like ours.

### 2.2 The role of emotional persona in multilingual academic contexts

When students write in multiple languages, the emotional persona they project in their academic work may vary depending on the cultural profiles shaped by their education and societal norms. These cultural imprints affect how they express emotions, assert authority, and engage with their audience, leading to different rhetorical choices and linguistic styles across languages. Kaplan (1966) and Cheng (1993) both explored how cultural thought patterns influence the structure of written discourse in the Chinese language, but they offered complementary insights into the topic. Kaplan proposed that Chinese writing often follows a circular or spiral thought pattern, characterized by indirectness and the gradual development of ideas. He suggested that Chinese students build their arguments by revisiting themes from different perspectives, which contrasts with the linear and thesis-driven structure typical in Western academic writing, such as writing in English. Cheng (1993), however, nuanced Kaplan’s view by showing that while circularity and digressiveness are present, especially in introductions and conclusions, Chinese writing also incorporates linear elements. Cheng (ibid.) found that Chinese students use both deductive and inductive structures in body section types (i.e., initial / end and middle parts), resembling Western styles of argumentation in certain contexts. This blend of circular and linear approaches reflects the influence of both cultural traditions and modern academic conventions on Chinese students’ writing. In his study of academic texts by L2 students from various linguistic backgrounds, Hinkel (2002) found notable differences in writing styles, influenced by students’ first languages and cultural conventions. Chinese and Korean students often displayed more indirect argumentation, while Arabic speakers used elaborate, repetitive structures. Spanish-speaking students, instead, tended to write with more personal, subjective tones. These variations sometimes reflect the influence of different rhetorical traditions on L2 writing, indicating the challenges students face in adapting to English academic norms, particularly in terms of clarity and structure. Building on Kaplan’s foundational ideas, Connor (1996) expands them by illustrating how English academic writing tends to be more linear and explicit in argumentation, while other cultures, such as Japanese or Arabic, might favor a more indirect or circular approach to presenting ideas.

Linguistic features can reveal specific aspects of writing cultures. Fløttum (2012) highlights notable differences in author visibility across academic writing in English, French, and Norwegian. English writers tend to use “I” more often, resulting in greater author presence and a more interactive style, where the writer frequently serves as a guide for the reader. In contrast, French academic writing employs the pronoun “on” (equivalent to “one” in English), which produces a more detached and abstract tone. Kruse et al. (2016) conducted an extensive analysis of academic writing in various European countries, offering valuable insights into the cultural and rhetorical factors shaping students’ approaches. This broader exploration helps explain why students from different countries adopt diverse writing strategies, including the use of personal pronouns, stance, and hedging techniques.

From a contrastive rhetoric perspective, the Romanian writing style is a mixed type, sharing similarities and differences with other writing cultures. A study by Chitez and Kruse (2012) shows that Romanian academic writing is shaped by traditional educational practices that emphasize memorization and literature-based genres such as *comentariul literar* (literary commentary) and *analiza literară* (literary analysis). These genres foster formal, detailed argumentation, particularly in response to literary texts, which aligns with the country’s teacher-centered system. However, educational policy shifts, influenced by the Bologna process, have introduced internationally recognized genres like the opinion essay, posing challenges for students as they adapt to new writing norms without sufficient guidance. This evolution mirrors broader trends in Romanian writing culture, where traditional, national-specific genres are increasingly blending with global academic standards. The same has been demonstrated by Băniceru et al. (2012), highlighting the evolving influence of Anglo-Saxon writing norms on traditional Romanian academic writing. While Romanian writing was historically shaped by French academic models, focusing on descriptive elements and form, recent shifts reflect the adoption of more structured, concise, and reflective practices typical of Anglo-Saxon conventions. However, the transition is incomplete, as Romanian writing still prioritizes descriptive moves over critical analysis, suggesting a partial and mechanical integration of Western academic writing trends. In terms of linguistic features distinguishing Romanian natives’ writing in Romanian versus English, several observations have been made. A corpus-based study by Bercuci and Chitez (2023) revealed that Romanian academic writing exhibits distinct linguistic traits that influence student writing, particularly when transitioning between Romanian and English. These include a preference for impersonal constructions and avoidance of first-person pronouns, which reflect a formal academic register. Romanian students tend to rely on descriptive and historicizing structures, frequently using phrases like “one of the most” and “at the same time,” which are common in Romanian academic traditions. Additionally, the frequent use of prepositions (such as “de,” “in,” and “la” – “of, “in,” and “to/at”) and formulaic expressions indicates a focus on description and formality rather than argumentation. These features often carry over into English writing, where students struggle to adapt to the more concise, argumentative, and personal style expected in Anglo-Saxon academic norms. From an emotional persona perspective, such features are associated with formality, detachment, and indirect expression. This tendency may stem from cultural and educational traditions that prioritize respect for authority and objective reporting over direct, personal involvement in arguments. Consequently, Romanian students often show hesitancy in asserting personal opinions or taking ownership of their ideas, contrasting with the more assertive, individualistic style of English academic writing. This culturally rooted linguistic behavior can lead to challenges in achieving argumentative clarity and critical engagement when writing in English. However, no corpus-based analysis focusing exclusively on the emotional features of Romanian students’ academic writing has been conducted.

In this context, the validation of the LIWC dictionary (Dudău and Sava, 2021, 2022) for use in academic research is a valuable tool, as it allows for detailed linguistic comparisons between Romanian and English texts. By enabling researchers to systematically analyze language use across these two languages, this validated dictionary supports the exploration of key linguistic features such as emotional tone, formality, and complexity.

### 2.3 Discipline-specific and genre-specific emotional identity traits

Academic writing is not only influenced by socio-cognitive factors and language- or culture-specific rhetorical traditions but also by the disciplinary and genre conventions that shape how knowledge is communicated within a field. Numerous studies have shown that the disciplinary epistemologies, communication patterns, and discursive practices differ from discipline to discipline (for instance, Langer and Applebee, 1987; Bazerman and Paradis, 1991; Monroe, 2002; Poe et al., 2010; Thaiss and Myers Zawacki, 2006). The variation in disciplinary conventions is due to differences in knowledge production, rhetorical goals, and audience expectations (Hyland, 2004). In hard sciences, writing is objective, concise, and data-driven, focusing on clarity and empirical evidence (Varttala, 2001), while humanities and social sciences make use of figurative language and demonstrate deeper engagement with sources to create emotional resonance and nuanced meaning (Machin and Mayr, 2012). Varttala (2001) also found that the use of hedging, or cautious language, varies across different disciplines, including economics, medicine, and technology. Citation practices also differ, with scientific fields favoring concise references to current research (Hyland, 1999), and humanities offering extended commentary on sources (Swales, 1990).

However, in point of the emotional approach to writing, numerous recent sentiment analysis studies have identified a generalizing trend called linguistic positivity bias, first discussed in research by Vinkers et al. (2015), which explored the use of positive and negative words in scientific PubMed abstracts between 1974 and 2014, showing that positive language increased more rapidly than negative language. In line with this, Xiao et al. (2023), who examined the evolution of sentiment in academic writing in China across the humanities and social sciences over time, found a noticeable shift towards more positive sentiment in recent decades. A study by Chen (2024) confirmed the distinct tone of medical writing, particularly in how it conveys emotions such as trust, hope, and surprise when addressing groundbreaking discoveries or unexpected findings. These emotions are subtly embedded through careful word choice – positive framing is used to highlight successful outcomes, while more cautious or measured language is employed when discussing study limitations, creating a balance between excitement and professionalism in medical discourse.

Specific emotion-signaling linguistic strategies are also genre-specific. The work of Swales (1990, 2004) pioneered the analysis of research genres and made the language of research accessible to scrutinized study. Swales work was a milestone in the study of research genres and in introducing methods from applied linguistics to the study of English as a research language. His corpus approach has been picked up by other researchers like Hyland (2000, 2005, 2008, 2009, 2012), who engaged in systematic corpus studies on such issues as metadiscourse, citation signals, praise and criticism, power and authority, use of “I”/“we.” This type of research is complementary to the concept of emotional persona in academic writing, as it examines how linguistic choices, such as personal pronouns, tone, and metadiscourse, reveal the writer’s emotional engagement, confidence, or detachment in scholarly discourse.

Previous research has shown that the linguistic cues present in academic writing provide valuable insights into how emotions such as positivity, enthusiasm, uncertainty, or confidence are conveyed within academic discourse. These cues offer a deeper understanding of the writer’s academic experience. Ultimately, academic writing is shaped by the interaction of cognitive processes, personal emotions, and the social and cultural norms of the academic community. Analyzing large linguistic datasets allows researchers to identify patterns of emotional expression and the rhetorical strategies employed by specific groups of writers.

## 3 Method

### 3.1 Corpus

For this study, the source of student writing was ROGER, a bilingual corpus of academic texts collected in 2018–2021 within nine Romanian universities (Chitez et al., 2021). As depicted in the ROGER platform (Strilețchi et al., 2022), the corpus contains 1,139 texts in English and 911 in Romanian, spanning various genres and being written by students at the Bachelor’s, Master’s or PhD degree levels across eight disciplines. The ROGER corpus was selected because it captures real-world academic writing produced by Romanian students across diverse disciplines, academic levels, and genres, thereby enhancing the generalizability of findings to a broader context of Romanian academic discourse. Initiated in 2017, it was the first bilingual Romanian-English learner corpus of this nature (Oravițan et al., 2022). To the best of our knowledge, the ROGER corpus offers a unique resource for studying academic writing within the Romanian context.

To reduce the class imbalances in genre and discipline, which could bias the results of the data analysis due to the overrepresentation of certain categories, we preprocessed these two categorical variables. In this vein, genres were grouped into two main categories: *(1) coursework and analytical writing*, encompassing essays, literary analyses, reviews, summaries, reading notes, assignments, tutorials, paragraphs, portfolios, CVs, interviews, and letters; *(2) research and academic papers*, comprising research papers, reports, Bachelor’s theses, Master’s theses, projects, and project documentations. This distinction was meant to separate reflective or summarizing tasks that allowed for more personalized language from formal, more standardized academic writing, which is typically used in research papers or other specialized materials. Similarly, the discipline variable was reduced from eight to three categories by combining the texts from computer science, engineering, and mathematics into *STEM*, those from political science, social science, economics, and law into *social sciences* while keeping *humanities* as a standalone class.

### 3.2 Automatic language analysis

#### 3.2.1 Tools and linguistic variables

To extract linguistic content and style from the ROGER texts, we used Linguistic Inquiry and Word Count 2015 (LIWC2015). The original English version developed by Pennebaker et al. (2015) was applied to the English texts, while the Romanian adaptation (Ro-LIWC2015; Dudău and Sava, 2021, 2022) was used for the Romanian texts. LIWC2015 is a closed-vocabulary text analysis tool consisting of a piece of software capable of determining the percentage of words in the input texts based on over 90 grammatical and psychological categories defined in a so-called *dictionary*, a list of labeled words, word stems, and emoticons established through rigorous research. The English LIWC2015 dictionary (Pennebaker et al., 2015) contains 6,549 entries, while the Romanian one includes 47,825. This difference in length is due to the particularities of Romanian in terms of morphology, semantics, and diacritics compared to English. Nevertheless, validation studies for RO-LIWC2015 (Dudău and Sava, 2021, 2022) have demonstrated that both dictionaries produce comparable results, indicating compatibility across languages.

From the multitude of LIWC2015 variables, we selected the following subset, which we considered most relevant to the goals of the current study: (1) first- and second-person pronouns (*i*, *we*, and *you* categories), as they indicate where the communication is directed—whether self-centered, toward a group with which the author identifies, or addressed to another person(s); (2) several parts of speech—*articles*, *prepositions*, *adverbs*, *conjunctions*, and *adjectives*—that suggest the degree of elaboration or complexity in the texts’ structure; (3) *verbs*, which show the extent to which the texts are action-oriented; (4) *positive* and *negative emotions*, as indicators of affective valence; (5) *family* and *friend* categories, illustrating a focus on close social relationships; (6) cognitive processes—*insight*, *causation*, *discrepancy*, *tentative*, *certainty*, and *difference*—that outline the depth of thinking; (7) motivational drives—*affiliation*, *achievement*, *power*, *reward*, and *risk* – that reflect key forces guiding behaviors or perspectives; (8) time orientation, showing whether the texts focus on the *past*, *present*, or *future*; (9) personal concerns—*work*, *leisure*, *home*, *money*, *religion*, and *death* – that reveal the presence of topics related to major life domains.

#### 3.2.2 Text selection and final dataset

Since there is no universally established minimum word count for valid text analysis with LIWC2015, we initially adopted the criteria used by Boyd and Schwartz (2021) to test the psychometrics of the LIWC-22 dictionary. Accordingly, we selected texts from the ROGER corpus that contained at least 100 words and had at least 65% of the words covered by the LIWC2015 dictionary (in English or Romanian, depending on the language of the text). However, applying these criteria resulted in the exclusion of about 27% of the Romanian texts, many of which were written in highly specialized language.

Excluding such a large portion of texts could have disproportionately affected the representation of certain genres or disciplines, potentially undermining the validity of our dataset. Therefore, to retain more valuable data without compromising the quality of the analysis, we adjusted the coverage threshold to 60% while maintaining the 100-word minimum. This adjustment allowed us to include 88.6% of the Romanian ROGER corpus and 98.3% of the English ROGER corpus, ensuring that a sufficient portion of each text’s linguistic data was analyzed for meaningful results. The final dataset for our study, following this selection, is presented in Table 1.

**Table 1:** Composition and linguistic characteristics of the final ROGER subset used in this study.

Composition	English corpus	Romanian corpus
Number of texts	1,120	807
Discipline		
Stem	368	37
Social sciences	474	214
Humanities	278	556
Genre		
Coursework and analytical writing	770	628
Research and academic papers	350	179
LIWC2015 tokenizer statistics		
Word count—*m*(*sd*)	1,782.51 (3,870)	1,374.32 (2,587.42)
Words per sentence—*m*(*sd*)	25.19 (10.18)	25 (10.70)
Dictionary coverage—*m*(*sd*)	83.14% (6.43)	69.48% (4.95)

As Table 1 indicates, there was a notable difference in LIWC2015 dictionary coverage between the English (83.14%) and Romanian (69.48%) texts. This difference might be attributed to a combination of factors, but the most prominent one could be that English was a foreign language for most of the students who wrote the ROGER texts (approximately 94% of the selected texts were written by Romanian students). Therefore, they may have used simpler, more general vocabulary, which is better represented in the LIWC2015 dictionary. In contrast, the Romanian texts, written in the students’ native language, may contain more specialized or nuanced academic terminology, which is likely less covered by the Romanian LIWC2015 dictionary.

Moreover, as shown in Table 1, the ratio between the English and Romanian texts is roughly 1.4 to 1, a moderate imbalance that would not necessarily require special attention during data analysis. In contrast, there were significant imbalances by genre and discipline, and we analyzed the linguistic markers associated with these two variables using a different approach, as explained in Section 3.3.

### 3.3 Data analysis strategy

To uncover the linguistic style and psychological contents in student academic writing and to reach more nuanced interpretations, we adopted a three-pronged approach, with each dimension complementing the others: (1) distinguishing between Romanian and English in student writing; (2) exploring the interactions between linguistic features; and (3) uncovering linguistic patterns. Throughout these analyses, we used different machine learning and statistical methods to provide multiple perspectives and deepen our understanding of student academic writing. Additionally, where appropriate, we applied cross-validation to manage the bias-variance trade-off, thereby improving the reliability and generalizability of our interpretations. Given that ROGER is a bilingual corpus, we performed within-language standardization before any data analysis. Specifically, for each LIWC2015 variable, we computed *z*-scores based on the mean and standard deviation of each language subsample, as suggested by previous research on multilingual data (Dudău and Sava, 2021; Meier et al., 2018). The following paragraphs provide detailed explanations of these technical aspects.

For the first objective—testing whether there are linguistic differences between Romanian and English languages in student writing—we applied two machine learning algorithms: *logistic regression* and *random forest*. Both addressed the classification problem of detecting language (English versus Romanian) based on the linguistic style and psychological contents assessed with LIWC2015. We started with logistic regression because it is a widely used and interpretable classification method that effectively detects linear relationships between the predictor variables and a binary outcome. Then, we built a random forest model, as this algorithm, by growing multiple de-correlated decision trees and averaging their predictions (Breiman, 2001; Hastie et al., 2009), is able to capture potential non-linear relationships between input and output and complex interactions between the linguistic features.

To ensure the robustness of the classification models, we implemented cross-validation for two purposes: to test the models on unseen data and to tune the random forest model. Specifically, we employed a stratified train-test split, selecting 75% of the data for training and 25% for testing while preserving the proportion of Romanian and English texts in both subsets. After the split, we performed within-language standardization on the LIWC2015 variables in the training subset. The *z*-scores were calculated separately for each language subset, using the mean and standard deviation of the respective subset. The same transformation was then applied to the test subset (i.e., the z-scores for the test subset were computed based on the means and standard deviations on the training subset to prevent data leakage and keep the test data exclusively for assessing the model performance).

For tuning the random forest model, we used 10-fold cross-validation and two accuracy metrics – area under the ROC curve (AUC) and F1-score – to evaluate the performance of different hyperparameter combinations. This cross-validation method involved dividing the training subset into ten equal folds, training the model on nine folds, and validating it on the remaining fold. The process was repeated ten times, with each fold used once as the validation set. We focused on four hyperparameters: the number of trees in the forest, the number of LIWC2015 features randomly selected at each split, the minimum number of texts in a leaf, and the maximum number of leaves. For each hyperparameter, we defined a search space: the number of trees ranged from 100 to 1,000, the number of predictors from 1 to 33, the node size from 1 to 20, and the maximum nodes from 10 to 100. A random search method, iterating over 500 combinations of these hyperparameters, was employed to identify the optimal combination based on the highest mean accuracy in the 10-fold cross-validation process.

After building the machine learning models on the training subset, we assessed the classification accuracy on the test subset. In this regard, multiple parameters were computed—accuracy, sensitivity (true positive rate), specificity (true negative rate), F1-score, and AUC. The higher these values, the better the classification accuracy. For AUC, clear benchmarks exist to aid in interpretation: AUC values between 0.50 and 0.70 are generally considered to show low accuracy, values between 0.70 and 0.90 indicate moderate accuracy, and values above 0.90 suggest high accuracy (Akobeng, 2007). In our study, achieving at least moderate classification accuracy suggested the presence of notable linguistic differences between the English and Romanian corpora, with higher accuracy indicating more pronounced distinctions.

To address our second data analysis objective—exploring the interactions between linguistic features—we conducted a network analysis using the 33 LIWC2015 variables as nodes. Before implementing this approach, we performed within-language standardization. Network analysis is particularly valuable when elements of interest can be viewed as components of a system where each is connected to others (Borsboom et al., 2021). Considering that natural language consists of words linked through semantic, morphological, and syntactic rules, which might resemble a system, network analysis can provide a novel perspective on student writing through the lens of linguistic features.

Specifically, to model the relationships between LIWC2015 variables and identify key linguistic interactions, we estimated a Gaussian graphical model using graphical LASSO regularization combined with the extended Bayesian information criterion (EBIC) for *edge* selection, following guidelines from Epskamp et al. (2018). This approach produces a parsimonious network where edges represent partial correlations between variables, accounting for all other variables in the analysis. The choice of LASSO regularization with EBIC was made to ensure that our network focused on the most prominent linguistic connections, balancing interpretability with accuracy. While this method has high specificity, meaning it effectively removes non-existent edges, it may be less sensitive in detecting true edges (Epskamp and Fried, 2018). Given the exploratory nature of this approach to academic writing, we prioritized interpretability, even if it meant potentially excluding some true edges. After estimating the network structure, we computed four centrality measures—betweenness, closeness, strength, and expected influence—to identify the most influential linguistic features in the network, providing insights into how these features interact and shape student writing. Finally, we assessed the stability of the network using bootstrap methods.

To meet the third and final data analysis objective – uncovering linguistic patterns across genres and disciplines—we applied k-means clustering, an unsupervised learning algorithm. The same set of 33 LIWC2015 categories was used as input variables for this analysis, preceded by within-language standardization to ensure comparability between the English and Romanian corpora. Specifically, k-means clustering allowed us to explore whether distinct types of texts emerged based on their linguistic features. To determine the optimal number of clusters, we used the majority rule method, testing solutions with 2 to 15 clusters. This method evaluates several cluster validity measures and recommends the number of clusters supported by the majority of these indices (Lesmeister, 2015). The clusters were built based on 1,000 random starting sets. Ultimately, to uncover potential linguistic differences across genres and disciplines, we applied the Chi-squared test to examine whether the cluster distribution was significantly associated with the texts’ genres and disciplines.

All analyses described in this section were performed using R and RStudio. Data manipulation and visualization were carried out using the *tidyverse* package (Wickham et al., 2019). The stratified train-test split was implemented with the *caTools* package (Tuszynski, 2021). Logistic regression was performed using the *glm* function from R’s base package, while the random forest model was trained and evaluated within the *mlr* framework (Bischl et al., 2016). Network estimation, visualization, and description were facilitated by the *qgraph* package (Epskamp et al., 2012), while network stability was assessed using the *bootnet* package (Epskamp et al., 2018). For k-means clustering, we used R’s built-in *kmeans* function from the *stats* package, in conjunction with the *NbClust* package (Charrad et al., 2014) for determining the optimal number of clusters.

## 4 Results

### 4.1 Distinguishing between Romanian and English languages in student writing

Altogether, the two classification models—logistic regression and random forest – used to differentiate between academic texts written in English and Romanian, based on the 33 LIWC2015 features, revealed complex distinctions between the two corpora.

Specifically, the performance of the logistic regression model on the test data was poor. Predicted probabilities for language classification were nearly constant and consistently below 0.50, leading to the misclassification of all texts as Romanian. This resulted in a low AUC of 0.45, which falls well below the commonly accepted threshold of 0.70 for acceptable classification accuracy. On the training subset, although the model’s intercept was significant (*β* = −0.33, *SE* = 0.05, *z* = −6.15, *p* < 0.001), all predictor variables had *p*-values of 1, indicating no significant contribution to the model. The null deviance (1964.8) and residual deviance (also 1964.8) suggest that the inclusion of the LIWC2015 predictors did not significantly improve the model over a null model. Multicollinearity was not a major concern, as most variance inflation factor (VIF) values were below 5, except for two variables: *verbs* (VIF = 6) and *focus on the present* (VIF = 5.24). However, these values are still not alarming, as VIF values below ten are generally considered acceptable (Bowerman et al., 2015; Field, 2018), and some scholars suggest that even higher values may not justify the exclusion or preprocessing of some variables (O’brien, 2007). These findings suggest that any linguistic differences between the Romanian and English texts, if present, were likely subtle or involved non-linear relationships, which logistic regression cannot capture effectively.

In line with this observation, the random forest model, which is better suited for detecting complex and non-linear patterns, displayed excellent classification accuracy on the test subset, with performance parameters close to 1, as depicted in Table 2. The tuning process yielded the optimal parameters of 716 trees, 2 predictors randomly selected at each split, a minimum node size of 7, and a maximum of 94 terminal nodes.

**Table 2:** Performance metrics for logistic regression and random forest models in detecting the language of texts.

Model	Accuracy	Sensitivity	Specificity	F1-score	AUC
Logistic regression	42%	1	0	0.59	0.45
Random forest	99%	1	0.99	0.99	0.99

The results were obtained using the test subset (*n* = 482), with “Romanian” as the positive class.

The top 10 most relevant linguistic features distinguishing Romanian from English student writings were the word frequencies for *death*, *home*, *family*, *religion*, *I*, *friend*, *we*, *you*, *money*, and *leisure*. Table 3 presents the entire feature hierarchy based on the *mean decrease Gini* value, which indicates how important each feature was in reducing the impurity of the trees in the random forest model, with higher values reflecting greater importance.

**Table 3:** Importance of LIWC2015 features in the random forest model for identifying the language of texts, with feature-level means and standard deviations (prior to standardization).

Random forest model			Descriptive statistics—*m*(*sd*)	
Hierarchy of LIWC2015 features		Mean decrease gini	Romanian texts	English texts
1.	Death	86.47	0.17 (0.35)	0.20 (0.43)
2.	Home	80.72	0.12 (0.23)	0.51 (1.33)
3.	Family	70.31	0.13 (0.33)	0.22 (0.46)
4.	Religion	64.74	0.24 (0.59)	0.24 (0.57)
5.	I	58.33	0.18 (0.43)	1.44 (2.27)
6.	Friend	54.53	0.15 (0.21)	0.20 (0.38)
7.	We	37.06	0.33 (0.42)	1.11 (1.60)
8.	You	35.21	0.26 (0.32)	0.63 (1.21)
9.	Money	19.52	0.76 (1.21)	1.12 (1.55)
10.	Leisure	18.55	1.02 (1.16)	0.99 (1.25)
11.	Focus on the future	12.38	0.46 (0.45)	1.03 (0.96)
12.	Risk	9.20	0.92 (0.80)	0.76 (0.81)
13.	Discrepancy	9.12	1.49 (0.93)	1.79 (1.40)
14.	Work	8.17	4.32 (2.46)	4.51 (3.46)
15.	Negative emotions	7.00	2.12 (1.47)	1.49 (1.18)
16.	Focus on the past	6.84	5.96 (2.18)	2.42 (1.60)
17.	Positive emotions	6.68	3.49 (1.62)	3.07 (1.56)
18.	Reward	6.06	1.05 (0.84)	1.19 (0.82)
19.	Achievement	5.60	2.87 (1.69)	2.00 (1.22)
20.	Tentative	5.45	3.07 (1.33)	2.49 (1.23)
21.	Articles	5.38	4.61 (1.35)	9.41 (2.53)
22.	Affiliation	5.38	1.58 (1.10)	2.33 (1.80)
23.	Verb	4.96	12.40 (2.28)	1.19 (0.82)
24.	Insight	4.91	4.04 (1.56)	2.75 (1.21)
25.	Prepositions	4.89	13.80 (1.77)	14.60 (1.79)
26.	Conjunctions	4.84	4.81 (1.52)	6.33 (1.40)
27.	Causation	4.83	3.75 (1.46)	2.58 (1.20)
28.	Focus on the present	4.62	6.68 (1.86)	8.66 (2.63)
29.	Difference	4.59	2.98 (1.22)	2.78 (1.21)
30.	Power	4.54	3.67 (1.81)	2.98 (1.57)
31.	Adverbs	4.52	6.30 (1.75)	3.50 (1.34)
32.	Certainty	4.26	2.00 (0.84)	1.46 (0.78)
33.	Adjectives	4.15	7.03 (1.77)	4.78 (1.40)

The results were obtained using the training subset (*n* = 1,445), with “Romanian” as the positive class.

Notably, personal concerns (except *work*) and personal pronouns dominated the top 10 list, alongside the social categories *family* and *friend*. Although direct comparisons of word percentages between languages are not the primary focus of random forest models, we observed that, on average, Romanian texts contained far fewer first-person pronouns, both singular and plural (see Table 3). This might suggest less personal engagement in Romanian writing compared to English. Therefore, a simplified inference might be that Romanian texts tend to exhibit a more formal style than the ones written in English.

Given the nearly perfect classification accuracy of the random forest model, we decided to keep the Romanian and English corpora separate for the remaining analyses, allowing us to explore language-specific linguistic patterns in greater depth.

### 4.2 Exploring the interactions between linguistic features in student writing

**Figure 1:**
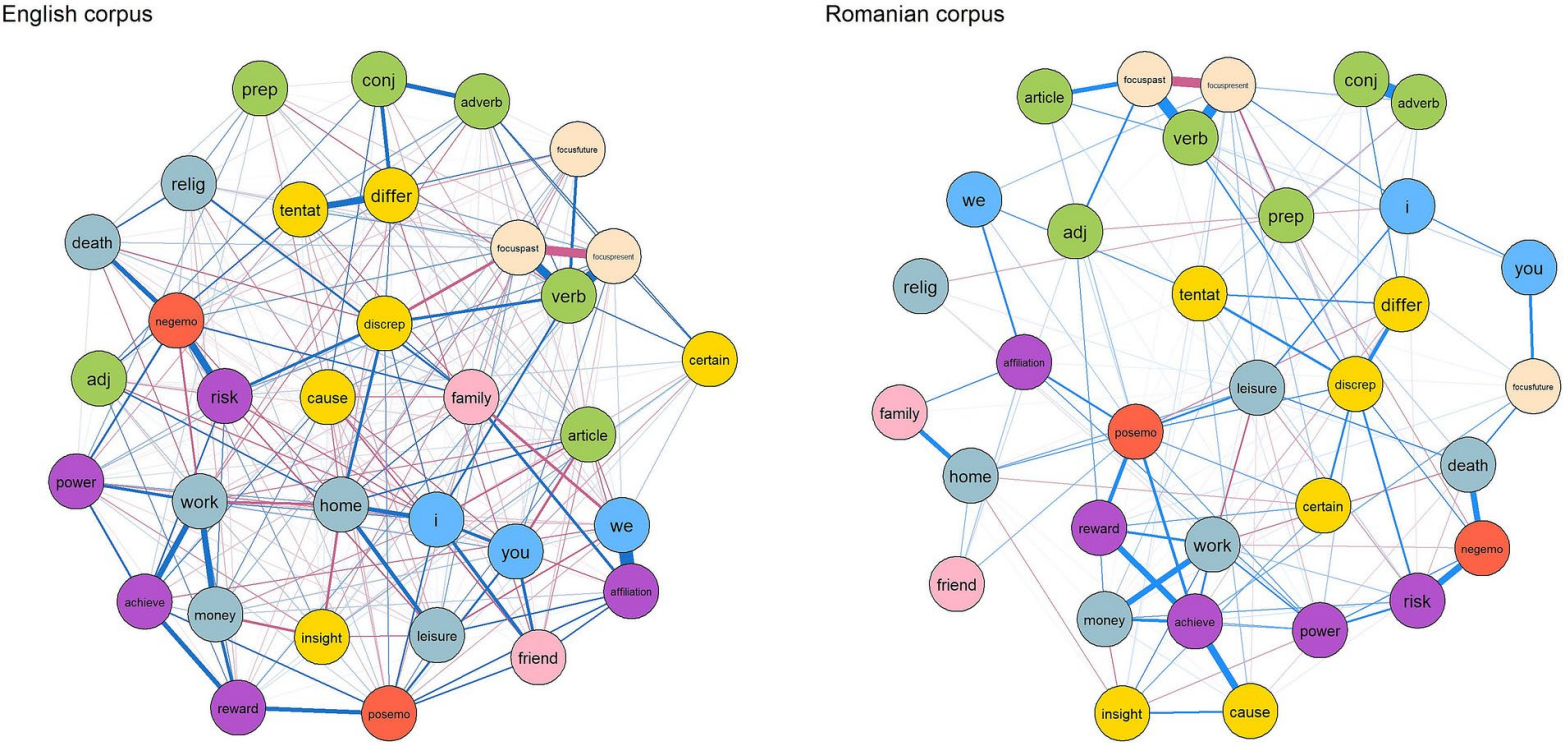
Estimated network structures of English and Romanian corpora using LIWC2015 variables. Edges in blue represent positive connections, while red edges indicate negative connections. The nodes are colored according to high-order categories—personal pronouns, other function words, affect, cognitive processes, social categories, drives, time orientation, and personal concerns.

To further investigate the relationships between linguistic features in student writing, we conducted separate network analyses for the Romanian and English corpora, utilizing the 33 LIWC2015 features as nodes in the network, as discussed in section *3.3 Data analysis strategy*. The goal was to identify how linguistic features interact and contribute to the overall structure of student writing. Figure 1 provides the visual representation of the two parsimonious networks of partial correlations between linguistic variables.

As observed, the network for the English texts displayed higher interconnectedness than the Romanian network, which was confirmed by the degree centrality metric. Degree centrality reflects the number of connections (or direct relationships) each node has. Specifically, in the English corpus, the number of connections per node ranged from 12 to 23, with the *power* category showing the highest number of connections, while *achievement*, *certainty*, and *money* had the fewest. In contrast, the Romanian corpus network exhibited fewer connections per node, ranging from 3 to 14. The second-person pronoun (*you*) category had the fewest connections, whereas *discrepancy*, *focus on the present*, *money*, *positive emotions*, and *work* had the highest number of connections.

**Figure 2:**
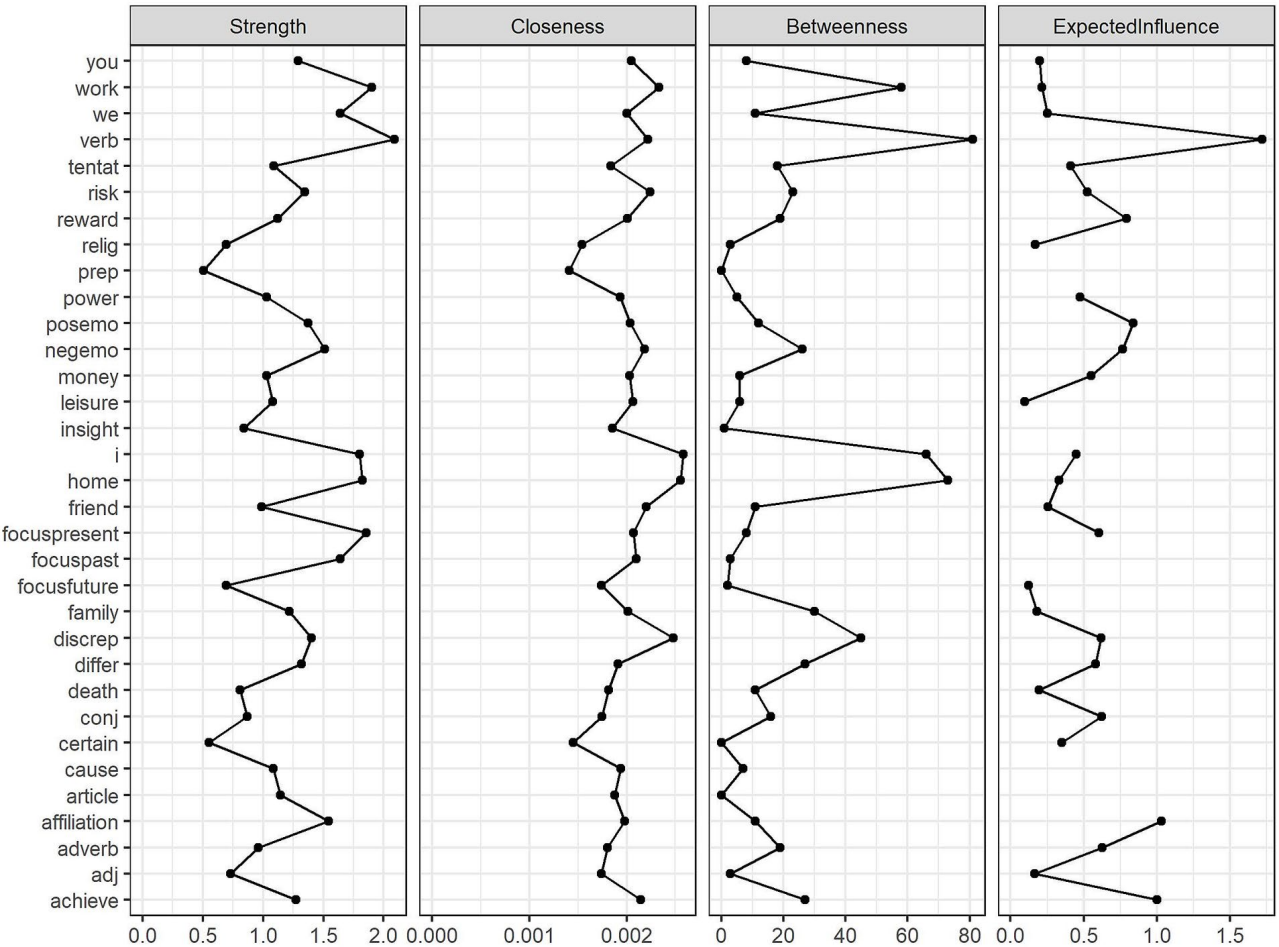
Centrality metrics of nodes in the English corpus network.

**Figure 3:**
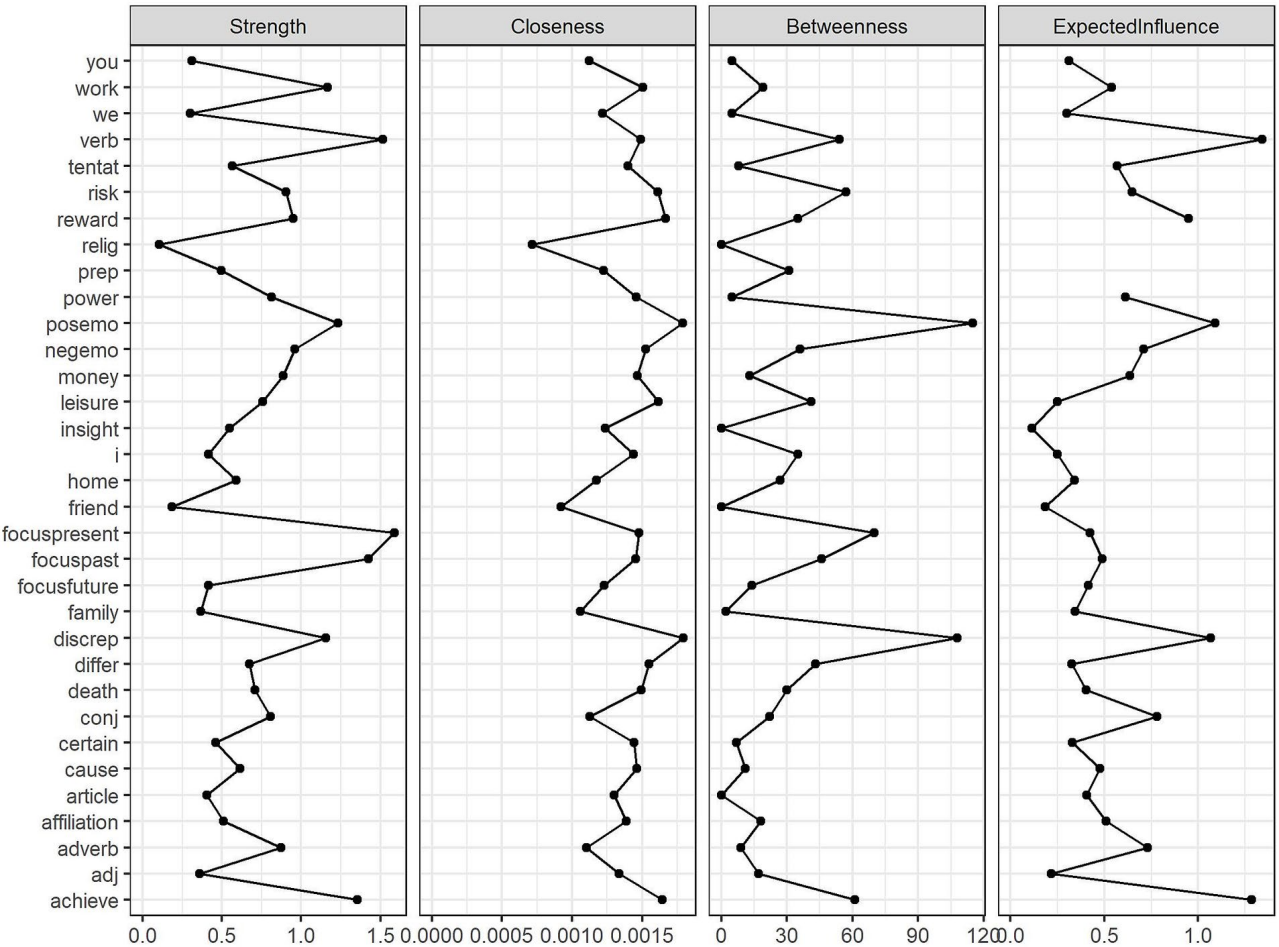
Centrality metrics of nodes in the Romanian corpus network.

To gain further insights into the linguistic interactions within each corpus, we computed four additional centrality metrics: strength (the sum of the absolute edge weights connected to a node), closeness (the inverse of the sum of all distances from a node to all other nodes, with higher values indicating closer proximity to the entire network), betweenness (how often a node lies on the shortest path between any two other nodes), and expected influence (a measure similar to strength but taking into account the direction of connections, with negative correlations reducing the influence of a node) as defined by Deserno et al. (2022). Figure 2 presents the results for the English corpus, and Figure 3 shows the corresponding results for the Romanian corpus.

For the English network, the strength metric revealed that *verbs* played a pivotal role in shaping the structure of English texts, strongly connecting to other linguistic categories. Other linguistic variables that could directly affect or be affected by many writing characteristics were *work*, *focus on the present*, *home*, and *first-person singular pronouns*. Similarly, the expected influence metric showed that *verbs*, *affiliation* drive, *achievement* drive, *positive emotions*, and *reward* drive emerged as the most influential variables, shaping the overall structure of the linguistic network. According to the closeness metric, the use of *first-person singular pronouns*, words referring to *home*, *discrepancy*, *work*, and *risk*, as well as the frequency of *verbs,* ensured information flow within the network, having a high probability of being easily affected when another linguistic feature changed in the network. Additionally, betweenness values were highest for *verbs*, *home*, *first-person singular pronouns, work*, and *discrepancy*, indicating that these linguistic features served as key connectors, bridging otherwise disparate elements in writing and facilitating transitions between different ideas or topics.

As far as the Romanian network was concerned, *focus on the present*, *verbs*, *focus on the past*, *achievement*, and *positive emotions* had the highest strength, indicating that action- and present-oriented language was about as central in Romanian writing as in the English texts. Likewise, *verbs* led in expected influence, followed by *achievement*, *positive emotions*, *discrepancy*, and *reward*. The least peripheral linguistic categories in the Romanian corpus network were *discrepancy*, *positive emotions*, *reward*, *achievement*, and *leisure*, meaning they acted as bridges between various linguistic features. The high closeness of *discrepancy*, in particular, may suggest that Romanian student writing, like English writing, contains nuanced or contrasting language to transition between ideas. The other variables high in closeness might indicate that the flow of information in Romanian student writing might be sustained mostly by addressing positive topics. In terms of betweenness, the linguistic categories measuring the focus on *positive emotions*, *discrepancy*, *present* time, *achievement*, and *risk* were prominent, suggesting that removing these linguistic markers would significantly disrupt the connections between other linguistic features, further highlighting their bridging role in Romanian academic writing.

**Figure 4:**
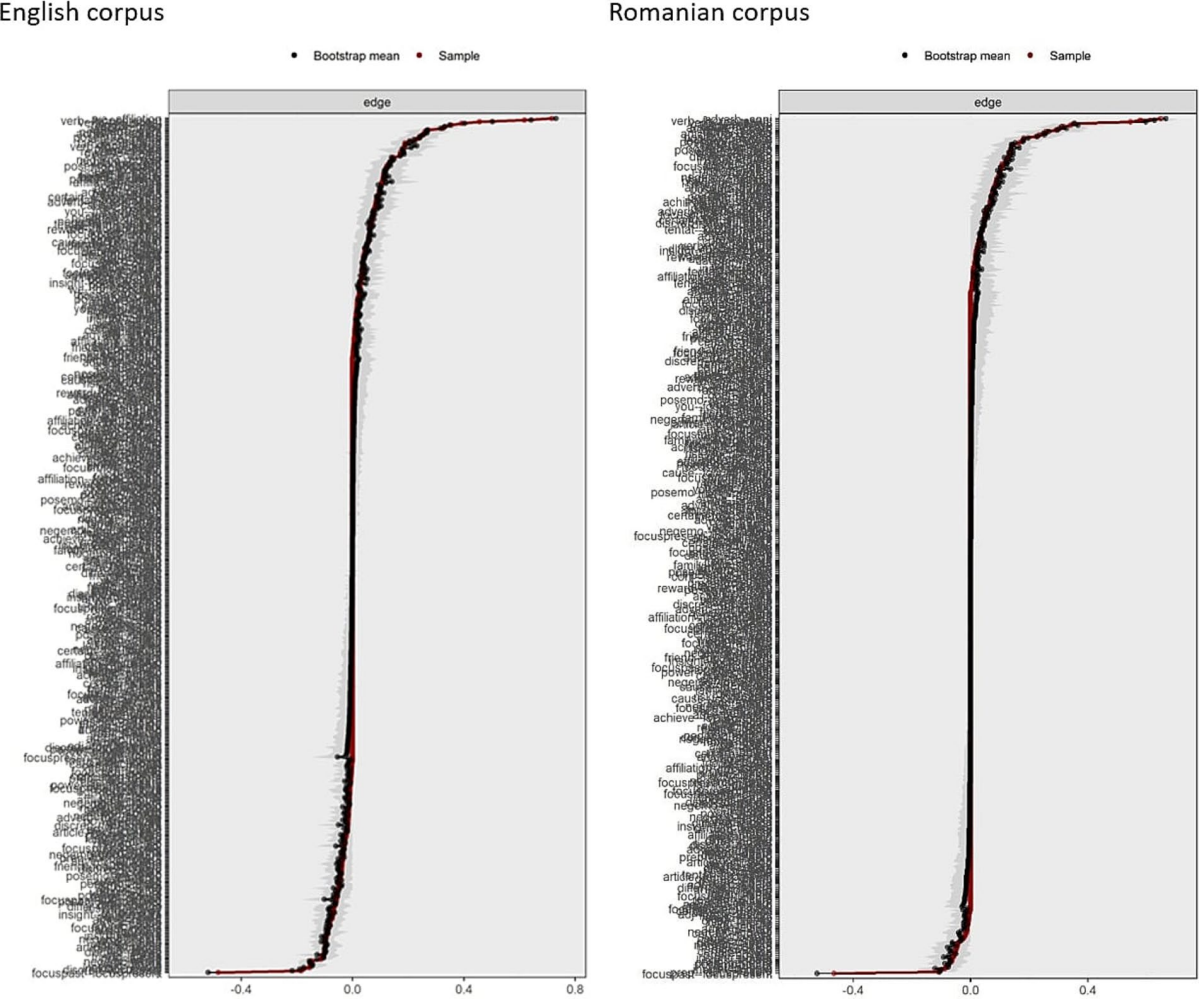
Bootstrapped confidence intervals of estimated edge-weights in the English and Romanian corpora networks of LIWC2015 features.

**Figure 5:**
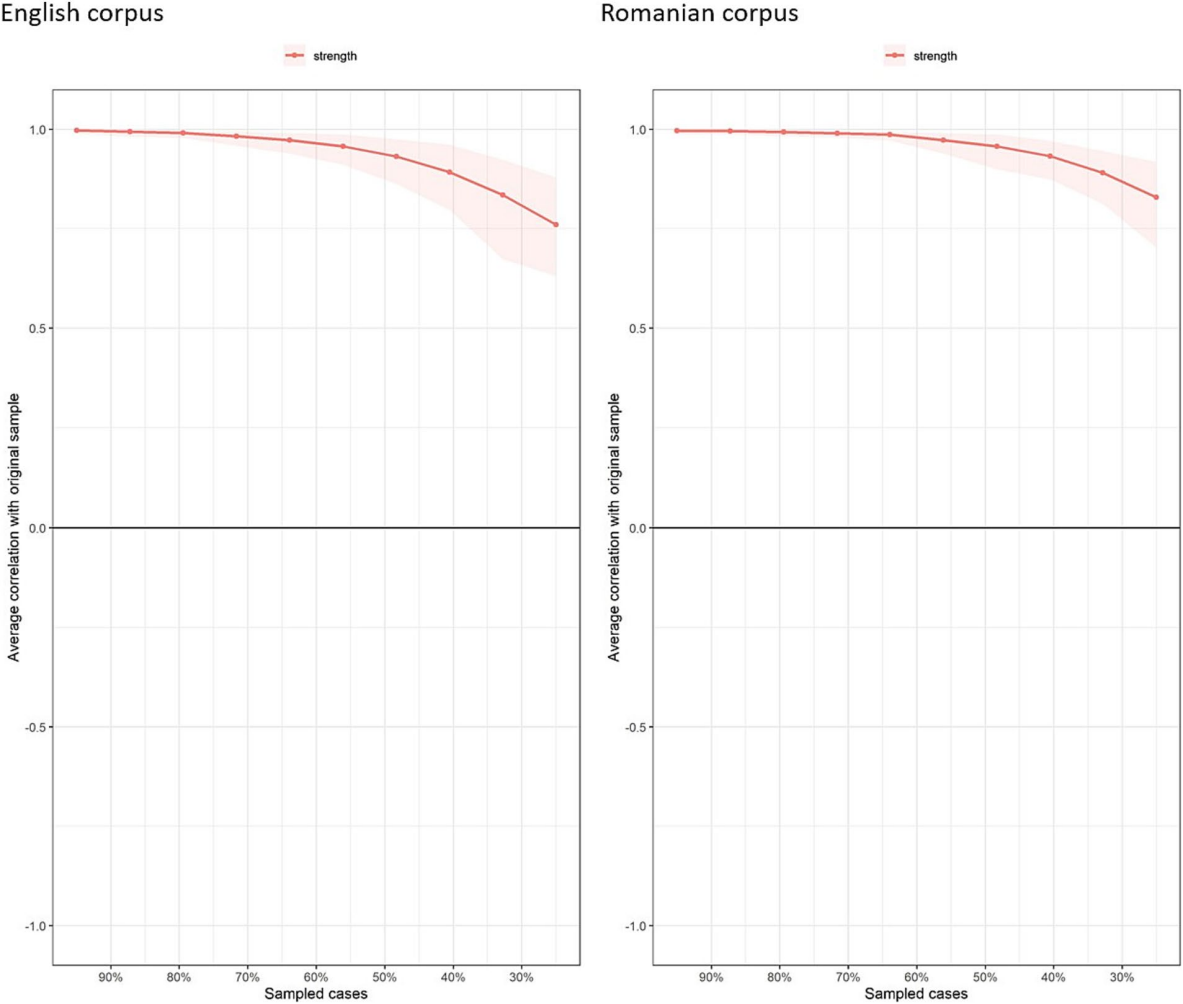
Case-dropping bootstrap results for strength centrality in the English and Romanian networks.

After the network estimation step, we used two bootstrap methods to assess the stability of the estimated networks. Specifically, we computed 95% confidence intervals for the edge weights (see Figure 4). Overall, both networks demonstrated multiple strong and reliable connections with narrow confidence intervals, though some edges were weak or potentially unstable. The case-dropping bootstrap method, which evaluates how the network structure changes when portions of the data are removed, was applied to assess the stability of the strength centrality across the networks. This method showed that even when up to 50–70% of the data was excluded, the strength centrality measures remained highly correlated with the full-sample estimates (see Figure 5).

### 4.3 Uncovering the linguistic patterns in student writing across genres and discipline

At the final stage of our data analysis, we applied the k-means clustering algorithm followed by the Chi-square test to examine whether distinct linguistic markers, based on the 33 LIWC2015 categories, organized the texts into meaningful groups and whether these groups varied by genre and academic discipline. The analysis was conducted separately for the English and Romanian corpora, using 1,000 random starting sets. According to the majority rule method, the English corpus was best represented by two clusters, whereas the Romanian corpus was represented by three.

Table 4 outlines the characteristics of these clusters through the mean *z*-scores for each LIWC2015 category. Although the within-language standardization procedure made the variables lose their original meaning (word percentages), we decided to use it before the k-means clustering, too, because Romanian and English might inherently have different linguistic distributions for certain LIWC2015 categories due to grammatical particularities and our interest lay in the linguistic patterns shaped by psychological or cultural factors in student writing.

**Table 4:** Centroids for LIWC2015 categories by cluster in English and Romanian corpora.

	English corpus		Romanian corpus		
Linguistic features	Cluster 1 *n* = 499	Cluster 2 *n* = 621	Cluster 1 *n* = 313	Cluster 2 *n* = 145	Cluster 3 *n* = 349
Function words					
I	0.38	−0.30	0.36	−0.18	−0.25
We	0.41	−0.33	0.19	0.07	−0.2
You	0.40	−0.32	0.14	−0.1	−0.08
Articles	−0.51	0.41	0.22	0.02	−0.21
Prepositions	−0.29	0.23	−0.48	0.39	0.27
Adverbs	0.28	−0.23	0.52	−0.26	−0.36
Conjunctions	0.22	−0.18	0.44	−0.14	−0.34
Verbs	0.60	−0.48	0.6	−0.08	−0.51
Adjectives	0.04	−0.03	−0.01	0.26	−0.1
Affect					
Positive emotions	0.40	−0.32	0.22	0.93	−0.58
Negative emotions	−0.15	0.12	0.5	−0.19	−0.37
Social domain					
Family	−0.21	0.17	0.15	−0.12	−0.08
Friend	0.19	−0.15	0.19	0.25	−0.27
Cognitive processes					
Insight	0.08	−0.06	−0.1	0.27	−0.02
Cause	0.15	−0.12	−0.26	1.02	−0.19
Discrepancy	0.56	−0.45	0.42	0.23	−0.47
Tentative	0.37	−0.30	0.23	0.24	−0.3
Certainty	0.35	−0.28	0.2	0.18	−0.25
Difference	0.29	−0.23	0.29	−0.42	−0.08
Drives					
Affiliation	0.37	−0.30	0.22	0.24	−0.29
Achievement	0.31	−0.25	−0.25	1.51	−0.4
Power	0.02	−0.02	0.12	0.78	−0.43
Reward	0.40	−0.32	−0.13	1.17	−0.37
Risk	0.19	−0.15	0.1	0.51	−0.3
Time orientation					
Focus on the past	−0.3	0.24	0.17	0.01	−0.16
Focus on the present	0.68	−0.54	0.49	−0.1	−0.4
Focus on the future	0.29	−0.24	0.18	0.32	−0.29
Personal concerns					
Work	0.31	−0.25	−0.39	1.26	−0.18
Leisure	−0.15	0.12	0.4	−0.22	−0.27
Home	0.03	−0.03	0.04	0.27	−0.15
Money	0.30	−0.24	−0.29	1.26	−0.26
Religion	−0.24	0.19	0.22	−0.22	−0.11
Death	−0.30	0.24	0.47	−0.36	−0.27

The table contains mean z-scores, which are not suitable for direct interpretations in terms of word percentages. English corpus contained 1,120 texts, while the Romanian corpus contained 807 texts.

As inferred from Table 4, distinct linguistic profiles emerged for the clusters in each language when comparing their centroids. A clear divide is present, especially between the two English clusters, one featuring more personal and emotionally expressive language and the other reflecting a more formal, structured style. Interestingly, the Romanian corpus exhibited a third cluster, which could reflect the more diverse academic writing styles in Romanian student texts.

In the English corpus, Cluster 1 was characterized by higher frequencies of personal pronouns and more words involving positive emotions, motivational drives, cognitive processes, and a preoccupation with work, money, home, and friendship. Moreover, this cluster was marked by the use of more verbs and a focus on the present and future, potentially indicating a more action- or goal-oriented approach. All these features, along with more adverbs, conjunctions, and adjectives, suggest a more expressive writing style potentially reflective of less formal academic texts and more personal engagement. Cluster 2 is the opposite of Cluster 1, scoring higher in categories such as articles, prepositions, and focus on the past, with lower use of personal pronouns and emotionally charged language. This indicates a more structured, formal writing style focusing on objective analysis and academic formality, even on topics such as family, religion, or death, which tend to be more specific to humanities or social sciences.

Regarding the patterns that emerged from the Romanian corpus, Cluster 1 stood out for higher frequencies in function words such as personal pronouns, articles, conjunctions, and verbs, as well as in linguistic markers of psychological complexity, as suggested by language referring to emotions (especially negative valence), cognitive processes (with a notable accent on discrepancies and differences), religion, and death. A preoccupation with leisure activities and family matters was also noticed. Thus, overall, the Romanian Cluster 1 was characterized by an emotionally expressive writing style. Cluster 2 was distinct due to its higher scores in LIWC2015 categories like positive emotions, insight, causation, achievement, power, reward, and risk. Moreover, it strongly focused on the future, work, and money, reflecting achievement-oriented or entrepreneurial themes, possibly denoting formal academic texts commonly found in social sciences such as economics, political science, or psychology. Finally, Cluster 3 showed a more disengaged and impersonal profile, with relatively low scores across categories, indicating a less distinctive, more moderate linguistic style that could represent general-purpose or mid-level academic writing.

The Chi-squared test results revealed significant associations between clusters and both genre and discipline in both English (χ^2^ = 156.46, *df* = 1, *p* < 0.001 for genre; χ^2^ = 184.96, *df* = 2, *p* < 0.001 for discipline) and Romanian corpora (χ^2^ = 175.11, *df* = 2, *p* < 0.001 for genre; χ^2^ = 295.73, *df* = 4, *p* < 0.001 for discipline). These results suggest that the linguistic patterns captured by the clustering process might be systematically related to the texts’ genre and academic discipline.

By examining the contingency statistics in Table 5 and the linguistic profiles discussed earlier in this subsection, it became apparent that the clusters emerged at the intersection between discipline and genre. This observation could further suggest that specific academic contexts or tasks might require distinct linguistic styles and contents, while disciplines might involve some internal variation in their approaches. For instance, the more personal and expressive cluster derived from the English corpus contained much fewer research and academic papers than the more formal cluster and, consistently, a high percentage of texts from social sciences (about 60% of the texts in Cluster 1). Similarly, many of the English STEM texts (44.8%) were distributed in Cluster 1, which aligns with the fact that a high proportion of papers within this discipline (65.5%) represented coursework and analytical writing. In the Romanian corpus, the three clusters reflected an even more diverse academic writing style. Cluster 1, characterized by emotionally expressive language, was linked to coursework and analytical writing and included a high proportion of humanities texts. In contrast, Cluster 2, marked by future orientation and achievement, aligned with the particularities of research papers and social sciences. Cluster 3 represented a general, more detached academic writing style, with very low representation from social sciences and a high concentration of humanities texts, suggesting a second type of coursework and analytical writing within this discipline. Additionally, a relatively high proportion of Romanian research and academic papers (33.5%) were also present in the disengaged-profile Cluster 3, further illustrating the complexity of writing styles within this corpus.

**Table 5:** Cluster distribution by discipline and genre in English and Romanian corpora.

	English corpus		Romanian corpus		
	Cluster 1 *n* = 499	Cluster 2 *n* = 621	Cluster 1 *n* = 313	Cluster 2 *n* = 145	Cluster 3 *n* = 349
Discipline					
Humanities	34	244	249	26	281
Social sciences	300	174	61	117	36
STEM	165	203	3	2	32
Genre					
Coursework and analytical writing	440	330	285	54	289
Research and academic papers	59	291	28	91	60

English corpus contained 1,120 texts, while the Romanian corpus contained 807 texts.

## 5 Discussion

### 5.1 Methodological novelty and linguistic insights

This study aimed to elucidate the emotional persona in university students’ academic discourse using LIWC2015, a powerful yet easy-to-use tool for automatic language analysis. Specifically, we sought to explore linguistic patterns across languages, genres, and disciplines, focusing on how multiple linguistic markers varied between English and Romanian academic writing. To this end, we relied on the ROGER corpus, which allowed us to contribute to a research niche regarding Romanian students. This is particularly interesting because Romania, as a former communist country, has undergone numerous socio-economic, cultural, and educational changes in the 35 years since the collapse of the communist regime.

A notable strength of this study lies in its reliance on a corpus collected entirely in Romania, ensuring that the differences observed between Romanian and English academic writing stem from participants within the same demographic and cultural context. Unlike comparative studies that examine texts produced in different countries, this research design allows for a more focused examination of how native versus second-language use interacts with cultural and psychological dimensions. In other words, this framework provides a unique opportunity to explore how linguistic choices in a second language (L2) may favor communication patterns characteristic of the target culture, such as the more direct and personalized style often associated with English, a language rooted in individualistic cultural norms.

Moreover, to gain a deeper understanding of the linguistic markers of academic writing, we implemented a complex data analysis strategy based on machine learning (supervised and unsupervised) and advanced statistical methods. In particular, the network analysis approach for examining how LIWC2015 variables were interconnected was an original choice that could also be valuable in other research contexts that involve this language analysis tool. Thus, our results might offer novel insights into how student writing reflects broader socio-cultural and academic conventions and how the specifics of academic language could be useful in academic writing pedagogy.

The very different classification accuracies of the logistic regression and random forest models highlighted the complexities involved in distinguishing between English and Romanian languages in academic writing based on LIWC2015 features. The logistic regression model performed poorly, indicating that if any linguistic differences between the two languages existed, they could not be captured well by a linear model. The random forest model, by contrast, achieved nearly perfect classification accuracy, suggesting that, indeed, the differences between the English and Romanian texts were significant, though subtle and non-linear or multi-dimensional. The top distinguishing LIWC2015 features in the random forest model – *death*, *home*, *family*, *religion*, *I*, *friend*, *we*, *you*, *money*, and *leisure—*suggested that the expression of personal engagement and writing about important life themes might differ between the two languages. The tendency toward fewer first-person pronouns in Romanian texts may indicate that students adopt a more formal, impersonal tone in academic writing in Romanian. Such a pattern could reflect differing cultural or educational expectations regarding academic discourse, where Romanian academic traditions might emphasize objectivity and detachment. In contrast, English academic writing may prompt more personal involvement and expression.

However, the lack of equivalence in discipline and genre representation across the two languages introduces an additional layer of complexity when comparing the two corpora. Specifically, the Romanian corpus contained a significantly higher proportion of humanities texts, whereas the English corpus included more contributions from STEM. This imbalance could have inadvertently diminished the observed linguistic differences, as the presumably more detached and impersonal nature of STEM writing in English and the more personal and emotionally expressive tone expectable from Romanian humanities writing may have diluted the formal and restrained style often associated with Romanian academic writing. Moreover, cross-linguistic differences might also have been underestimated due to the *foreign language effect*. Research suggests that thinking and writing in a non-native language can reduce the influence of emotions and encourage more logical, rational thinking (Circi et al., 2021; Hayakawa et al., 2022; Keysar et al., 2012). Thus, writing in English (L2) might require heightened cognitive control, leading to simplification or a shift toward rationality over emotional depth. However, it is noteworthy that the results did not reveal clear patterns of higher emotionality in the Romanian (L1) texts, suggesting that other psychological, cultural, or contextual factors may play a role in shaping the emotional personas when writing in these languages.

The network analysis approach revealed a distinction in the interconnectedness of linguistic features between the English and Romanian corpora, with the English corpus displaying higher overall connectedness, as evidenced by the greater number of edges. This might suggest that students tended to integrate various linguistic elements more cohesively when writing in English as a second language, potentially reflecting their adaptation to the linear, argument-driven structure typical of English-language academic discourse (Hinkel, 2002). Nevertheless, the centrality metrics showed that while both languages emphasize action-oriented and motivational language, the English network illustrated a more personal and self-reflective tone. In other words, Romanian writing remained more formal and detached, which aligns with previous research (Bercuci and Chitez, 2023).

The cluster analysis revealed two linguistic profiles within the English corpus and three within the Romanian corpus. A third cluster in the Romanian corpus could underscore more diverse academic writing in this linguistic context, which might reflect the transitional state of Romanian academic writing, where traditional genres and styles coexist with more contemporary, global academic conventions, as emphasized, for instance, by Băniceru et al. (2012) and Chitez and Kruse (2012). The clusters derived from the linguistic features were significantly associated with both genre and discipline. In the English corpus, Cluster 1, characterized by a more personal and expressive style, was predominantly composed of coursework and analytical writing, and social sciences texts. Cluster 2, which exhibited a more formal and structured style, was more heavily associated with research papers. Similarly, in the Romanian corpus, Cluster 1 contained more emotionally expressive language and was strongly linked to coursework and analytical writing, while Cluster 2, with its focus on achievement and future orientation, was more common in social sciences and research papers. Cluster 3 was characterized by a general, detached academic writing style, with a substantial concentration of humanities texts, a minimal representation from social sciences, and the inclusion of nearly all STEM texts.

This study represents a novel exploration of the emotional dimensions of Romanian academic writing, a field that has been largely neglected in prior research. By using the bilingual ROGER corpus, the first to comprehensively capture the state of university academic writing in Romania, we offer unique insights into the interplay between language, emotion, and academic conventions. The original dataset enables a bilingual comparative approach that highlights cross-linguistic differences and cultural nuances in academic discourse. Furthermore, the methodological approach employed in this research, i.e., integrating LIWC for automated emotional analysis, represents a groundbreaking advancement in Romanian academic writing studies. Unlike previous research, which focused primarily on structural or rhetorical features, this study introduces the psychological dimension by capturing the emotional persona embedded in student writing. By uncovering significant contrasts in emotional expression across languages and disciplines, our study not only enriches the understanding of Romanian academic discourse but also contributes to the broader field of multilingual academic writing. The analysis offers critical insights for developing culturally sensitive teaching methodologies that address the emotional and linguistic needs of students navigating multilingual academic environments.

### 5.2 Culturally shaped linguistic features of Romanian academic writing

The emotional persona of Romanian students’ academic writing emerges as a distinctive interplay of linguistic markers shaped by cultural, disciplinary, and contextual influences. Our study identifies several key features that distinguish Romanian students’ academic writing in L1 from academic writing in L2 English, drawing on the analysis of 33 LIWC2015 features and the structural relationships among these features.

As David’s (2015) comprehensive study on the psychological profile of Romanians highlights, the culture is characterized by a blend of emotional restraint, collectivism, and a tendency toward skepticism and indirect communication. These traits are deeply embedded in Romanian social norms, influencing academic traditions and communication styles.

As explained below, this profile is mirrored in students’ academic writing in Romanian. However, interestingly, when writing in English, students tend to express a different style, as though they adopt, at least partly, a different academic persona that struggles to depart from the Romanian-specific restraint style and embrace a more personal, direct, and expressive communication, which is in line with Anglo-Saxon conventions. This difference might illustrate the tension between culturally and educationally ingrained communication norms and the need to adapt to global academic standards.

Specifically, the analysis presented in the current study reveals that Romanian academic writing is characterized by a more formal, detached style, as evidenced by the significantly lower use of first-person pronouns compared to English texts. This trend reflects broader cultural norms in Romania, where academic traditions emphasize objectivity and deference to authority over personal engagement. In contrast, academic writing in L2 English displays greater use of personal pronouns and emotionally expressive language, indicating a shift toward the assertive and individualistic norms of Anglo-Saxon academic conventions. The random forest model results highlight the importance of linguistic markers related to personal concerns, such as “death,” “home,” and “family,” as well as personal pronouns like “I” and “we.” While these features are prominent in distinguishing between English and Romanian texts, their relative frequencies suggest a nuanced linguistic style in Romanian academic writing. For instance, Romanian texts often avoided direct references to the self, aligning with the cultural emphasis on collective expression and indirect communication. Network analyses further reveal distinct patterns of interaction among linguistic features in Romanian academic texts. Compared to L2 English writing, the Romanian corpus exhibited fewer connections between linguistic variables, indicating a less cohesive integration of elements. Key features such as “discrepancy,” “positive emotions,” and “focus on the present” emerge as central in shaping the structure of Romanian texts. These features serve as bridges, connecting otherwise disparate linguistic markers and facilitating the transition between ideas. This indicates that Romanian students rely on nuanced language to maintain flow and coherence in their writing, despite a generally formal and restrained emotional tone.

The cluster analysis provides additional insights into the diversity of writing styles in Romanian versus L2 English. In the English L2 corpus, two distinct clusters emerge: one characterized by a personal and expressive style and the other by a more formal and structured approach. The expressive cluster features a higher use of personal pronouns, positive emotion words, and markers of motivational drives, reflecting a goal-oriented and engaging tone. This style, often found in coursework and analytical writing, aligns with Anglo-Saxon academic norms that encourage individual expression and critical engagement. Conversely, the formal cluster, associated with research papers, is marked by higher frequencies of articles, prepositions, and a focus on past events, indicative of objective analysis and academic rigor.

In comparison, the Romanian corpus exhibits three clusters, highlighting greater diversity in writing styles. The first cluster, marked by emotionally expressive language, shares similarities with the English expressive cluster but includes a notable emphasis on negative emotional markers and cognitive processes such as “discrepancy” and “difference.” This suggests a more reflective and complex emotional engagement, particularly in less formal academic contexts like coursework. The second cluster, distinguished by future orientation and markers of achievement and power, aligns with the English formal cluster but exhibits stronger motivational themes, likely reflecting the influence of social sciences and research-oriented writing. The third Romanian cluster represents a detached and impersonal style, with low frequencies across most linguistic categories, reflecting a neutral tone often associated with general-purpose academic writing.

Such distinctions underscore the influence of cultural norms on academic writing. While English texts often reflect a balance between expressiveness and structure, Romanian texts exhibit a stronger separation between emotional engagement and formal academic norms. The additional cluster in the Romanian corpus suggests a transitional stage, where traditional academic expectations coexist with emerging global influences, creating a broader spectrum of styles. The comparison highlights the challenges faced by Romanian students as they adapt to bilingual academic expectations. The expressive styles in both corpora indicate a shift toward greater emotional engagement in less formal contexts, while the formal styles reflect ongoing adherence to disciplinary conventions. By understanding these patterns, educators can better support students in navigating the linguistic and cultural complexities of multilingual academic writing.

### 5.3 Pedagogical implications

Study findings offer several key takeaways for teaching practices at the university level, particularly in multilingual and multicultural academic settings. First, the distinct linguistic profiles identified in Romanian academic writing, ranging from formal and detached styles to emotionally expressive approaches, highlight the need for pedagogical strategies that address this diversity. Educators should recognize and accommodate the influence of cultural norms on writing, especially the preference for objectivity and formality in Romanian academic traditions. Tailored instruction can help students balance these norms with the more personal and assertive styles encouraged in English academic writing.

Second, the contrast between the cohesive, highly connected linguistic networks in English texts and the more segmented structure of Romanian writing suggests a need for targeted training in integrating linguistic elements cohesively. Workshops focusing on the use of connectors, cohesive devices, and argumentation strategies could bridge this gap, helping students produce writing that aligns with global academic expectations while maintaining their unique cultural perspective.

Finally, the findings on emotional personas in writing provide an opportunity to incorporate discussions of voice, tone, and audience into writing curricula. By encouraging students to explore how emotional engagement enhances clarity and persuasiveness in their texts, educators can encourage greater confidence in navigating different academic conventions. Addressing these issues explicitly in coursework could enable students to adapt their writing more effectively across genres, disciplines, and cultural contexts.

In sum, our research highlights the importance of a nuanced, culturally informed approach to teaching academic writing at the university level. By leveraging these insights, educators can support Romanian students in developing versatile, internationally competitive writing skills while respecting and integrating their linguistic and cultural heritage. This dual emphasis ensures that students are not only prepared to meet international academic standards but are also empowered to contribute their unique voices to the broader academic conversation.

### 5.4 Study limitations and prospects for future research

Given the dual challenges posed by Romania’s socio-historical context and the demands of multilingual academic writing, the current study sought to build on a critical gap in understanding how emotional personas are reflected in student writing. However, the topic of emotional persona in academic writing is complex, and as with any study, our research is not without its limitations, which present opportunities for further exploration and development.

First, certain methodological shortcomings warrant further exploration. In this regard, the data was limited to a sample of Romanian students from nine universities – all state institutions – which may affect the generalizability of the findings to other cultural or linguistic contexts or even to the population of Romanian university students. The ROGER corpus already offers broad coverage, but the sample could not be considered nationally representative. Similarly, our dataset was marked by several class imbalances, which could have introduced a confounding effect in all our findings, especially those related to network and cluster analysis. Moreover, the recoding process of the genre and discipline variables did not involve multiple raters or a rigorous methodology, which could have impacted the quality of the new classes.

To address these methodological challenges, replication studies and efforts to refine variable control are needed. Moreover, future studies could expand the scope to include students from different linguistic backgrounds, allowing for a more comprehensive understanding of how emotional personas are expressed across different languages and academic traditions. LIWC2015 could provide the technical means to extend the current research to a multilingual, intercultural context, revealing valuable insights into the emotional and cognitive aspects of academic writing. However, its closed-vocabulary nature may overlook some of the more nuanced or context-specific elements of student writing. Future research could also explore how open-vocabulary approaches, which allow for analyzing emergent linguistic patterns, could complement the findings based on LIWC dictionaries, which follow a list of predefined linguistic features.

Second, the main goal of the current study was to understand whether different emotional personas are present in Romanian versus English academic writing and whether discipline- and genre-specific linguistic patterns exist. This research topic, while valuable, opens the door to numerous related questions. For instance, while our study focused on Romanian students’ one-time written academic discourse, future research could explore how emotional personas evolve over time. Thus, a longitudinal perspective could bring a deeper understanding of how academic writing skills – and the emotional personas embedded within them – develop as students advance through their academic careers. An additional valuable question would be whether tailored pedagogical approaches could help students refine their emotional personas in academic writing and whether such refinements could foster improved communication, critical thinking skills, motivation, or cultural adaptability.

## 6 Conclusion

This study sheds light on the emotional and cognitive characteristics of Romanian (L1) and English (L2) student writing, revealing significant cross-linguistic, as well as discipline- and genre-specific patterns. By leveraging the LIWC2015 tool alongside machine learning and network analysis, we identified distinct linguistic profiles in the Romanian and English corpora. These results might suggest the role of the second language (L2) cultural norms in shaping academic writing and emotional expression. Our findings contribute to a deeper understanding of the complex interplay between psycholinguistic and cultural factors, offering valuable insights for educators and researchers in multilingual academic settings.

## Data Availability

The original contributions presented in the study are included in the article/supplementary material, further inquiries can be directed to the corresponding author.
